# Evolutionary Constraints Favor a Biophysical Model Explaining *Hox* Gene Collinearity

**DOI:** 10.2174/13892029113149990003

**Published:** 2013-06

**Authors:** Yannis Almirantis, Astero Provata, Spyros Papageorgiou

**Affiliations:** 1 National Center for Scientific Research “Demokritos”, Institute of Biosciences and Applications, 153 10 Athens, Greece;; 2 National Center for Scientific Research “Demokritos”, IAMPPNM – Division of Physical Chemistry, 153 10 Athens, Greece

**Keywords:** Chromatin, *Hox* collinearity, Limb bud, Invertebrate-vertebrate evolution.

## Abstract

The *Hox* gene collinearity enigma has often been approached using models based on biomolecular mechanisms. The biophysical model is an alternative approach based on the hypothesis that collinearity is caused by physical forces pulling the *Hox* genes from a territory where they are inactive to a distinct spatial domain where they are activated in a step by step manner. Such *Hox* gene translocations have recently been observed in support of the biophysical model. Genetic engineering experiments, performed on embryonic mice, gave rise to several unexpected mutant expressions that the biomolecular models cannot predict. On the contrary, the biophysical model offers convincing explanation. Evolutionary constraints consolidate the *Hox* clusters and as a result, denser and well organized clusters may create more efficient physical forces and a more emphatic manifestation of gene collinearity. This is demonstrated by stochastic modeling with white noise perturbing the expression of *Hox* genes. As study cases the genomes of mouse and amphioxus are used. The results support the working hypothesis that vertebrates have adopted their comparably more compact *Hox* clustering as a tool needed to develop more complex body structures. Several experiments are proposed in order to test further the physical forces hypothesis.

## INTRODUCTION

*Hox * genes is a class of genes belonging to the homeobox gene superfamily. The homeobox is a sequence of 180 base pairs that remains impressively invariant during evolutionary DNA reshufflings. Each* Hox *gene entails such a homeobox and it encodes a specific transcription factor. The family of these transcription factors plays a pivotal role in development and particularly in the morphological formations on the head to tail axis of the embryos. 

In 1978 E.B. Lewis discovered the phenomenon of *Hox* gene collinearity [1]. According to his observations in *Drosophila,* the *Hox *genes are activated following the sequential spatial order anterior-to-posterior on the embryonic primary axis. Surprisingly, these genes are located on the chromosome following the same order- in the direction from the anterior end (3’) to the posterior end (5’) of the DNA fiber. This astonishing correlation, coined *gene collinearity*, was later established as a universal feature observed in almost all animal species [2]. The evolutionary origin of this collinearity has been extensively studied [2-5]. During animal evolution the organization of *Hox* genes has taken divergent forms. In particular, it was assumed that tandem duplication of an ancestral *ur-Hox* gene and sequential evolutionary modifications lead to the generation of an organized gene array [3, 4]. Durston has recently proposed that posterior prevalence (the dominance of posterior *Hox* genes over anterior ones) plays a unique role to vertebrate evolution [5]. From the different forms of these *Hox* gene clusterings (from tight and ordered to loose or split), the vertebrate clusters are best organized in a short and compact form [2].

Vertebrates have four paralogous *Hox* gene clusters (*HoxA, HoxB, HoxC *and* HoxD*) each one located in a different chromosome. Every cluster contains a variable number of *Hox* genes, numbered as 1, 2, 3… up to 13, where counting starts from the anterior side of the chromosome. From the above 13 genes, in every cluster some genes are missing.

**Fig. (1):**
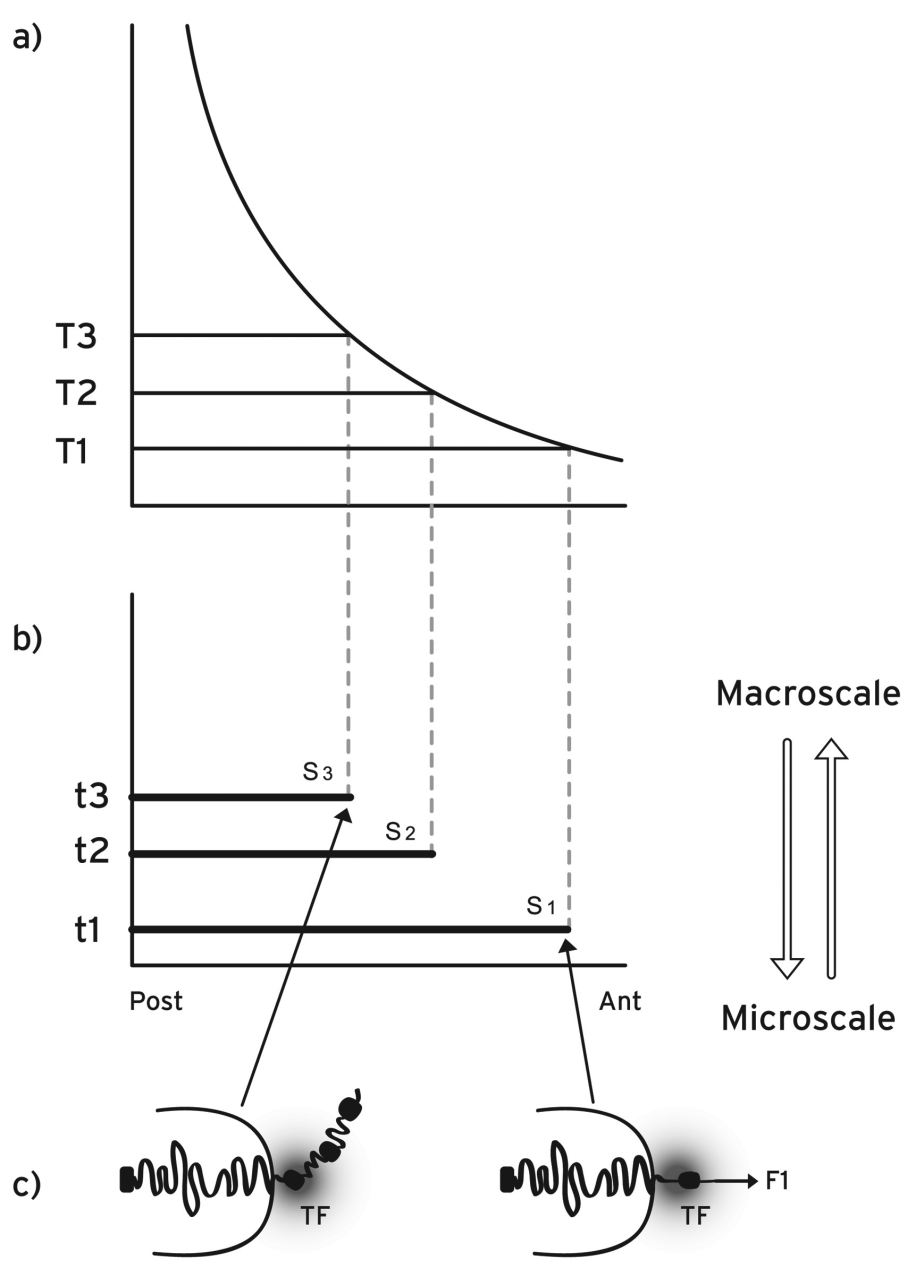
**Morphogen gradient (macroscopic scale) and *Hox* gene activation (microscopic scale) in space and time. a**) Concentration thresholds (T1, T2, T3,…) divide the anterior-posterior axis in partially overlapping expression domains. **b**) the time sequence (t1, t2, t3, …) together with (T1, T2, T3,…) determine the *Hox1, Hox2, Hox3,*… activation in space and time. S1, S2, S3,…are the partially overlapping and nested expression domains of *Hox1, Hox2, Hox3,*…**c**) in an anterior cell of S1, a small force **F1** pulls *Hox1* (black spot) out of the CT toward the ICD in the regime of the transcription factory TF (grey domain). At a later stage in a more posterior location S3, a stronger force (not shown) pulls *Hox3* out of the CT.

The above property of collinearity is more precisely defined as *spatial collinearity* [4] (Fig. **1**). Besides this *spatial collinearity*, it was established in vertebrates that the timing of gene activation follows a *temporal collinearity*: *Hox1* is activated first, then *Hox2* is activated followed by *Hox3 *and so on [6]. Furthermore, a third kind of collinearity was also observed in the vertebrates: when at a given location along the anterior-posterior axis several *Hox* genes are activated, the expression of the most posterior gene in the cluster is stronger compared to the expressions of the other more anterior genes (*quantitative collinearity*) [7]. Quantitative collinearity can be seen as the first step toward posterior prevalence [5]. 

During the last decade genetic engineering experiments have illuminated several features of the still enigmatic phenomenon of expression collinearity of the clustered *Hox* genes. In a series of experiments on the *HoxD *cluster in embryonic mice, members of this cluster were deleted or duplicated [8-10]. The results were impressive and initiated a deeper understanding of the *Hox* gene collinearity mechanism.

It is a challenge to understand the above combined experimental results. To this end, several models have been proposed most of them based on the well studied biomolecular mechanisms incorporating the action of enhancers, inhibitors and other genetic regulators [8, 9]. Although the proposed biomolecular models can describe several experiments, many other results remain ‘surprising’ [8] or ‘impossible to anticipate’ [9]. 

In a quite different approach, a “biophysical model” was first formulated in 2001 based on physical principles and in particular proposing the relevance of physical forces acting on *Hox* clusters [11]. Since then this model has been elaborated, completed and satisfactorily compared to the accumulating experimental data [12-16].

In the following we outline the main features of the biophysical model and present some recent experimental results that are unexpected according to the established biomolecular models. We then propose an explanation of these data in the framework of the biophysical model. Furthermore we present some evolutionary arguments, based on stochastic modeling, explaining why the differences in the form of *Hox* clustering between different animal taxonomic groups (e.g. arthropods, non-vertebrate chordates and vertebrates) favor the physical forces hypothesis of the biophysical model. In the last section we propose several experimental setups to test the biophysical model and we outline our main conclusions.

## THE BIOPHYSICAL MODEL FOR *HOX* GENE COLLINEARITY

The conceptual motivation for the formulation of the biophysical model was the observation of the multiscale nature of *Hox* gene collinearity. On the one hand the pattern along the embryonic anterior-posterior axis extends in a spatial (macroscopic) scale of the order up to 1mm. On the other hand the (microscopic) size of a typical *Hox* cluster is of the order of 500 nm [13]. The correlation of sequential structures in spatial dimensions differing by more than 3 orders of magnitude renders *Hox* gene collinearity a characteristic multiscale phenomenon (Fig. **1**). In order to deal with this multiscale coherence, Systems Biology seeks the implication of mechanisms originating from other disciplines like Physics and Mathematics [17].

**Fig. (2):**
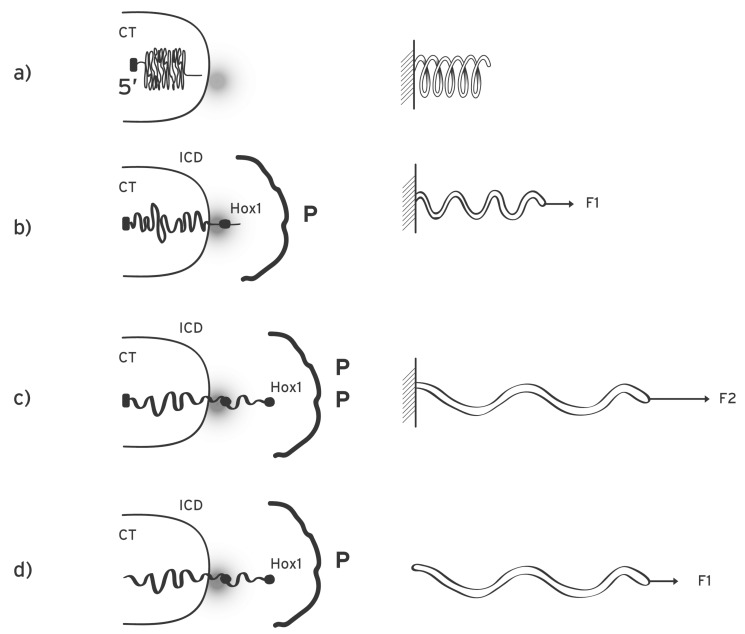
**Mechanical analogue of *Hox* cluster decodensation and extrusion**. **a**) Left: before activation the *Hox* cluster is condensed inside the chromatin territory (CT) at the posterior 5’ end. The transcription factory (TF) is represented by a grey domain. Right: mechanical analogue: an uncharged elastic spring is fixed at its left end. **b**) Left: the cluster is slightly decondenced and *Hox1* (black spot) is extruded in the interchromosome domain (ICD) in the area of the TF. The P-molecules are allocated opposite the cluster. Right: a small force **F1** is applied at the loose end of the spring and expands it slightly. **c**) Left: the cluster is further decondenced and the extruded *Hox2* is located in the transcription factory area. *Hox1* moves off the TF domain and its activation is reduced. Right: a bigger force (**F2** > **F1**) expands further the spring. **d**) Left: the posterior end of the cluster is cut-off. Right: the fixed end of the spring is removed and a smaller force (**F1**) expands and dislocates the spring as in Fig. **2c** (right).

According to the biophysical model for *Hox* gene collinearity during the early embryonic stages, a macroscopic morphogen gradient is generated along the anterior-posterior axis of the embryo with the peak of the gradient located at the posterior end of the embryo (or the distal tip of the secondary axis of a limb bud) [18] (Fig. **1**). Examples of such gradients are the Sonic hedgehog (Shh) or the Fiber growth factor (FGF) whose concentrations are graded along the axes of the developing limb [18]. The signals from such gradients are transduced inside the cells and the varying morphogen concentrations carry a positional information for every cell of the morphogenetic field. The transduced signals trigger the production of specific molecules P which are transported and allocated at specific positions inside the cell nucleus. Many examples of such molecules have been extensively studied (see e.g. [13, 19]). We assume these molecules are located in the neighborhood of the *Hox *cluster and their apposition is schematically depicted in (Fig. **2**). When the *Hox* genes are inactive the *Hox *cluster is sequestrated in a particular nuclear domain inside the chromosome territory where the genes are inaccessible to their transcription factors [11, 20]. In this ‘ground state’, where the P-molecules are absent, no force is exerted on the cluster (Fig. **2a**). The production and apposition of these molecules starts and a force F1 is created pulling *Hox1 *out of its niche (Fig. **2b**). This occurs in cells of domain S1 above morphogen threshold T1 up to time t1 (Fig. **1**). The production and apposition of P-molecules continues in domain S2 up to time t2 in cells where the morphogen is above threshold T2 while the pulling force increases to F2 [15, 16]. Thus spatial and temporal collinearities are naturally generated and the gene activation of *Hox1, Hox2, Hox3…* proceeds step by step and the expression domains are partially overlapping as shown in (Fig. **1**).

It was shown that, contrary to common belief, it is the DNA that moves toward the transcription factories where immobilized polymerases activate the genes [21]. Note that independently *Hox *gene activation was recently associated with DNA movement [10]. A translocated* Hox* gene leaves the Chromosome Territory (CT) and approaches the Transcription Factory (TF) in the Interchromosome Domain (ICD) (Fig. **2**). The basic hypothesis of the biophysical model attributes *Hox* gene movement to a physical force. How can such a force be generated? We assume the cluster itself is endowed with a property, denoted ***N***, which can combine with property ***P*** of the P-molecules allocated in the cluster environment. The two properties, ***P*** and ***N***, can combine to create a force **F** acting on the cluster with **F **depending linearly on both ***P*** and ***N***.

(1) **F = *P*** * ***N***

In the abstract equation (1) several interpretations of ***P*** and ***N*** are possible. Heuristically, the following picture will prove useful: ***N*** can represent a ‘negative’ charge of the cluster which is reminiscent of the negative electric charge of the DNA backbone. Furthermore, a complementary ‘positive’ charge ***P*** can be attributed to P-molecules. In equation (1), **F** resembles a quasi-Coulomb force where the relative distance between ***P*** and ***N*** is neglected. **F **pulls the cluster and its strength is weak when ***P*** is small e.g. in the anterior cells (Fig. **2b**). For more posterior cells (higher ***P***) the force is stronger (Fig. **2c**). When a gene moves away from the ‘factory’ the intensity of its activation drops sharply [21] and this causes naturally quantitative collinearity [15, 16]. Note that this approach is only formalistic since we do not know the real nature of ***P*** and ***N***. However, as we will show below, this formalism can successfully describe most results of genetic engineering experiments. Other possible force creating mechanisms were proposed elsewhere [11].

The action of **F **on the cluster can be suitably represented by a mechanical analogue of an expanding elastic spring [15, 16]. The anterior end of the spring (3’) is loose and moves from inside the CT toward the ICD. At the posterior end (5’), the spring is fixed inside the CT (Fig. **2**). The elastic properties of the cluster have not been studied in depth. However, a degree of reversibility was observed: after activation, *Hoxb1* is ‘reeled in’ toward the CT [22]. 

In the early developmental stages, the biophysical model predicts an entanglement of the *Hox* gene expressions in space and time following the rule: early expression is associated with anterior expression while late activation is linked with posterior expression [14, 15]. This interlocking agrees with the observed link of temporal collinear activation of *Hoxb* genes and their collinear spatial expression in the chick embryo [23]. At later stages of development an uncoupling is observed between space and time for the *Hoxd* expressions [9]. In this respect note that temporal collinearity is the key to understand *Hox* gene cluster organization in evolutionary terms [24]. It turns out that temporal collinearity is associated with the ordered and compact *Hox *gene clustering of vertebrates (see below). 

## GENETIC ENGINEERING EXPERIMENTS ON *HOX* CLUSTERS

In recent years several genetic manipulations have been performed in mice consisting mainly of *Hox* gene deletions, duplications or splittings of the gene clusters. These experiments are essential in exploring the mechanism responsible for *Hox* gene collinearity of vertebrates. Below we explain comprehensively the experimental results in terms of the biophysical model and compare them to the biomolecular model descriptions.

### Posterior Gene Deletions and Duplications

When a DNA region inside a cluster is deleted, the total “charge” ***N*** is accordingly reduced. The opposite occurs when a DNA region is duplicated. As an example, consider *a Hox* cluster and a probe gene whose *wild type* expression is compared to the expression of the same gene in mutant embryos where some other *Hox *genes were deleted. According to the biophysical model, the consequences of these deletions depend on the relative location of the deletions [14-16]*.*

#### a) Posterior deletions:

**Fig. (3):**
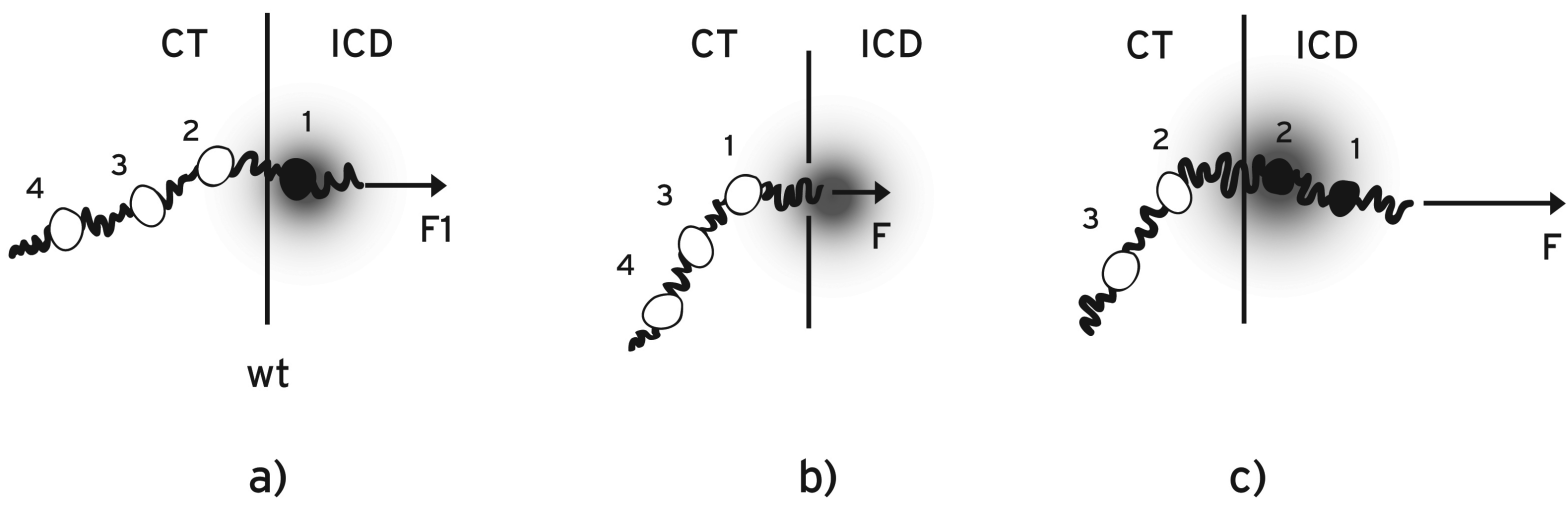
**Posterior *Hox* gene manipulations**. **a**) In the *wt Hox* cluster the posterior end (5’) is fixed but the anterior end (3’) is loose and a small force **F1** pulls *Hox1* (black disc) from CT to ICD in the region of the transcription factoty. **b**) When *Hox2* is deleted ***N*** diminishes and the force **F1** decreases to **F**. **F** does not suffice to extrude *Hox1* from the CT to the ICD. According to Eq. (**1**) (**F** = P*N) for the extrusion of *Hox1*, **F** must increase to **F1**(posteriorization). **c**) When *Hox2* is duplicated ***N*** increases. Hence a stronger force **F** pulls both *Hox1* and *Hox2* inside the ICD. *Hox1* moves away from the TF domain.

The deleted gene(s) are posterior to the probe gene (Figs. **3a,b**). In this case ***N*** decreases and in order to compensate in Equation 1 for the extruding force **F**, ***P*** must increase [14, 15]. Therefore, according to the biophysical model, it is expected a delayed posteriorization for the expression of the probe *Hox *gene. This expected result was indeed confirmed but it was ‘unexpected’ according to the established biomolecular models [8]. 

#### b) Posterior duplications

have an opposite effect (premature anteriorization) on a probe *Hox* expression (Fig. **3c**). Note that after a posterior duplication (increase of **F**) the probe gene is translocated further inside the ICD away from the CT/ICD border. This will cause a downregulation of the probe gene expression as a manifestation of quantitative collinearity [15]. This natural consequence, according to the biophysical model, was confirmed in the *Hoxd10* expression in the limb of mutant mouse embryos [25]. This observation cannot be reproduced by biomolecular mechanisms.

### Anterior Deletions

For the biophysical model, anterior gene manipulations are more complicated because the extruded fiber contributes to the modification of the pulling force [26]. 

**Fig. (4):**
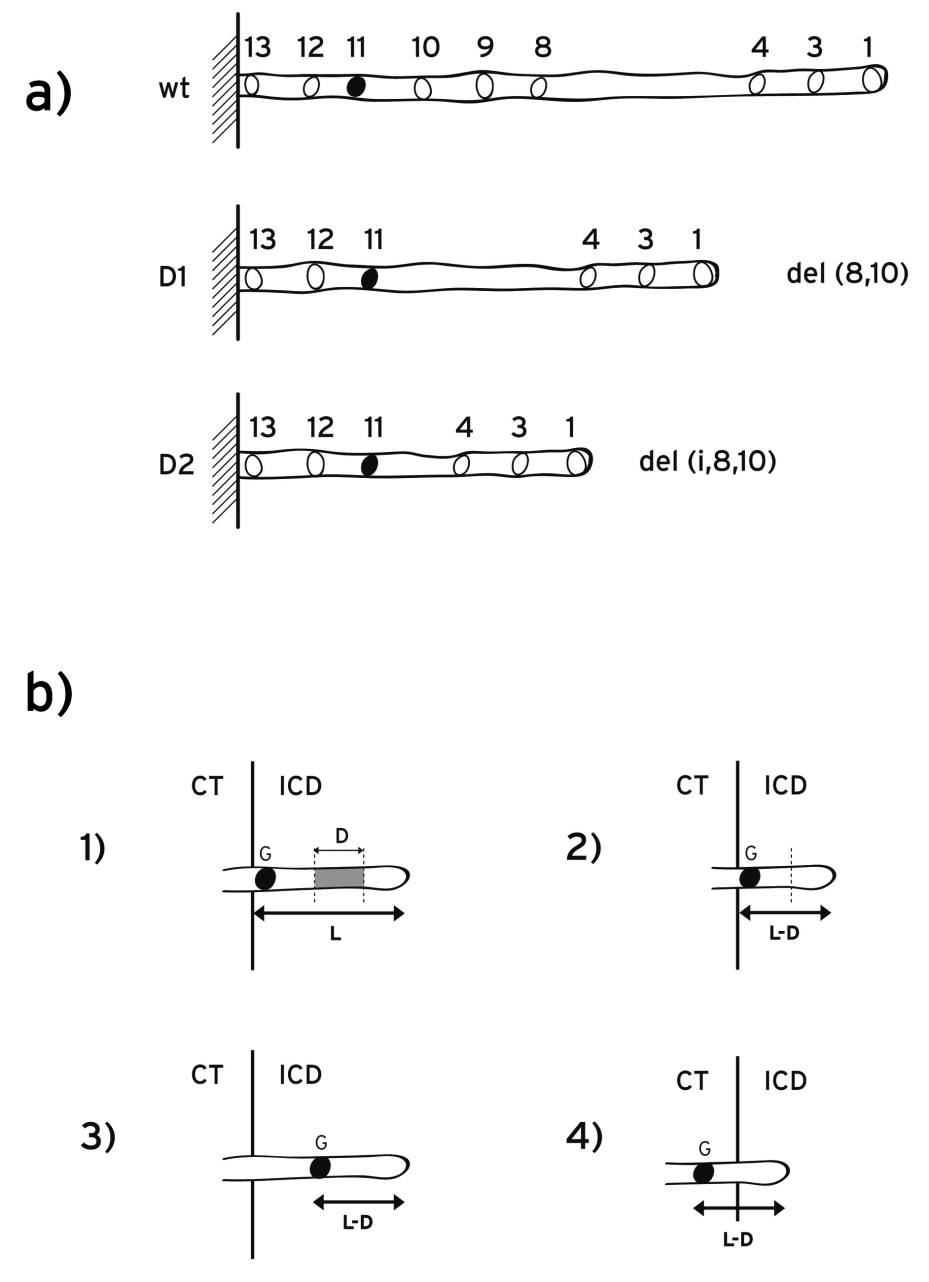
**Anterior *Hoxd* deletions**. **a**) (*xt*): Schematic representation of the *wild type HoxD* cluster and the probe gene *Hoxd11* (black disc). The length of the extruded fiber (from the anterior end to *Hoxd11*) is L. (D1): The anterior region D1 = [*Hoxd8*-*Hoxd10*] is deleted. (D2): The anterior region D2 = [*i*-*Hoxd8*-*Hoxd10*] is deleted. In D2, besides D1, the intergenic region (i) between *Hoxd4* and *Hoxd8* is deleted. **b**) Cases of anterior deletions. 1) Probe gene G moves from the CT where the gene is inactive toward the (ICD) where the gene is activated. The extruded fiber length of the *wt* probe gene is L (from the anterior end of the cluster to the probe G). D is an anterior DNA region to be deleted. 2) After the deletion D, the extruded DNA length is E where E = L-D: the probe gene G remains in the same position in ICD as in the *wt* case. 3) E > L-D. The extruded length E exceeds L-D. The probe gene moves further inside the ICD. For the activation of G, anteriorization is possible: G can retreat toward the CT/ICD border. 4) E < L-D. The probe gene remains inside the CT where its activation is not possible. G activation is possible only if G can move toward ICD (posteriorization).

The biophysical model for the early developmental stages, with some limited refinements, can explain the experimental data collected at somehow later stages. As an example consider the expression of *Hoxd11 *in *wild type* embryos and compare it to the expression of mutant embryos in which the anterior region [*Hoxd8*- *Hoxd10*] was deleted (Fig. **4**). The expression of the mutant embryo shows *wild type* spatial distribution [9]. In contrast, when the region [i-*Hoxd8*-*Hoxd10*] was deleted, the expression of *Hoxd11* was dramatically extended anteriorily. In deletion del(i*-*8*-*10), adjacent to del (8-10) the intergenic DNA fragment “i” was also deleted (Fig. **4**). “i” is located between *Hoxd4* and *Hoxd8*. These results were ‘impossible to anticipate’ by biomolecular models [9]. 

As reported recently in a 3D *in vivo* analysis of conformational chromatin modifications during *Hox* cluster activation, *Hox* genes move stepwise from a compartment where the cluster is inactive to a spatially distinct domain where *Hox* genes are transcriptionally active [10].

Combining the above experiments, it was found that the mutant *Hoxd11* with deletion del (8-10) was not ectopically expressed in the anterior trunk of the mouse embryo and it was not associated with the active part of the cluster. In contrast, the mutant *Hoxd11* with deletion del(i*-8-*10) was ectopically expressed in this anterior trunk and it was strongly associated with the active part of the *HoxD *cluster [10]. 

It is a challenge to understand the above combined experimental results. According to the biophysical model the property ***N*** (e.g. the negative charge) is distributed all over the cluster and a local deletion inside the *Hox* cluster will cause accordingly a decrease of ***N***. More specifically, an anterior DNA deletion affects a probe gene expression following two consecutive steps: a) the deletion D causes a reduction of ***N***, hence the normal pulling force **F** will be reduced to **F_c_.** b) a consequence of the weaker force **F_c_** is that the extruded DNA fiber is shorter than the *wild type (wt)* extruded fiber length (Fig. **4**). Suppose that the extruded length of the *wt *fiber from the anterior end of the cluster to the probe gene is L. The anterior deletion D will cause a shortening of this length to (L-D). Schematically this is depicted in (Fig. **4**). Consider now E the extruded fiber of the mutant probe gene due to **F_c_** (Fig. **4**). E and (L-D) are not necessarily equal because the elastic properties of DNA differ drastically from place to place along the chromatin fiber. E depends on these local properties of the DNA fiber since this fiber is strongly inhomogeneous [26].

There are three possible scenarios for mutant *Hoxd* expressions after an anterior gene deletion D (Fig. **4**).

#### 1 (L-D) = E.

The deleted region D causes an equal length reduction of the extruded gene fiber. (This is the case of uniform elastic properties along the whole DNA). For the gene whose expression is probed, the position in relation with the interface CT/ICD does not change (Fig. **4b** (**2**)).

#### 2 (L-D) < E.

The mutant extruded fiber E is longer than (L-D) and the gene whose expression is probed shifts inside ICD. A shorter fiber extrusion may suffice for the expression of this mutant gene (Fig. **4b** (**3**)). According to the equation (1) for **F** this shortening results from a weaker force resulting from a ***P ***reduction. The reduction of ***P*** leads to a premature anteriorization of this mutant gene expression as elaborated in detail in references [14, 15].

#### 3 (L-D) > E.

In this case, the mutant gene remains inside the CT and no activation of this gene will be observed (Fig. **4b** (**4**)). Activation of the mutant gene is possible only if G is translocated to the ICD. This can occur by a ***P*** increase (e.g. posteriorization).

It is interesting to look for actual manifestations of the above three cases. Although the biophysical model applies to the early developmental stages (up to about E9.5) the conclusions can be extended to later stages for a comparison with the existing experimental data.

- In Case 1 (E =L-D) the shortening of the extruded fiber equals the length of the deleted region so that the position of the mutant probe gene remains invariant compared to the corresponding *wt* probe gene (Fig. **4b**). This possibility can explain the observation of Tschopp *et al.* [9]: the mutant *Hoxd11* expression along the anterior-posterior axis after deletion del (8-10) is comparable with the *wt Hoxd11* expression.
- Case 2 (E>L-D) can describe all anterior deletions in the mouse limb [8]: for probe genes *Hoxd13*,* Hoxd11* and *Hoxd10 *and for different lengths of anterior deletions, the mutant expressions in stages E9 up to E10.7 are prematurely expanded anteriorily. In Case 2 could also belong the observed ectopic anteriorization of the *Hoxd11* expression on the anterior-posterior axis after deletion del(i*-*8-10) [9] (Fig. **4b**): the extruded fiber exceeds the length (L-D) and the mutant *Hoxd11 *moves inside the ICD. Therefore a retreat toward interface CT/ICD is permissible and an anteriorization of *Hoxd11* may occur for deletion del(i*-*8-10). Note that a ‘dramatic gain’ of the mutant *Hoxd11 *expression was noticed [9] and this is understandable since *Hoxd11* shifts toward the interface ICD/CT where the gene activation is stronger in the area of the transcription factory (quantitative collinearity).
- Case 3 (E<L-D) can explain the observed expressions of *Hoxd13* after anterior deletions (see Fig. **5 G**, **H**, **I** of ref. [9]): deletion del(10-12) leaves the expression of *Hoxd13* unchanged along the anterior-posterior axis compared to the *wt* expression. In contrast, the longer deletion del(9-12) leads to a strong suppression of *Hoxd13*. Note that *Hoxd13* is the most posterior gene of the cluster so that further posteriorization is impossible. Therefore, after deletion del(9-12) and according to the biophysical model, *Hoxd13* remains inside the CT area where it cannot be activated (Fig. **4b**). This is in agreement with the observed strong suppression of *Hoxd13 *expression [9]. On the one hand, the above analysis explains the *prima facie* unexpected expressions of mutant *Hox*d genes. On the other hand, it provides evidence supporting the hypothesis that physical forces cause the collinearity of *Hox* gene expressions.

### Posterior Splittings of the Gene Cluster

**Fig. (5):**
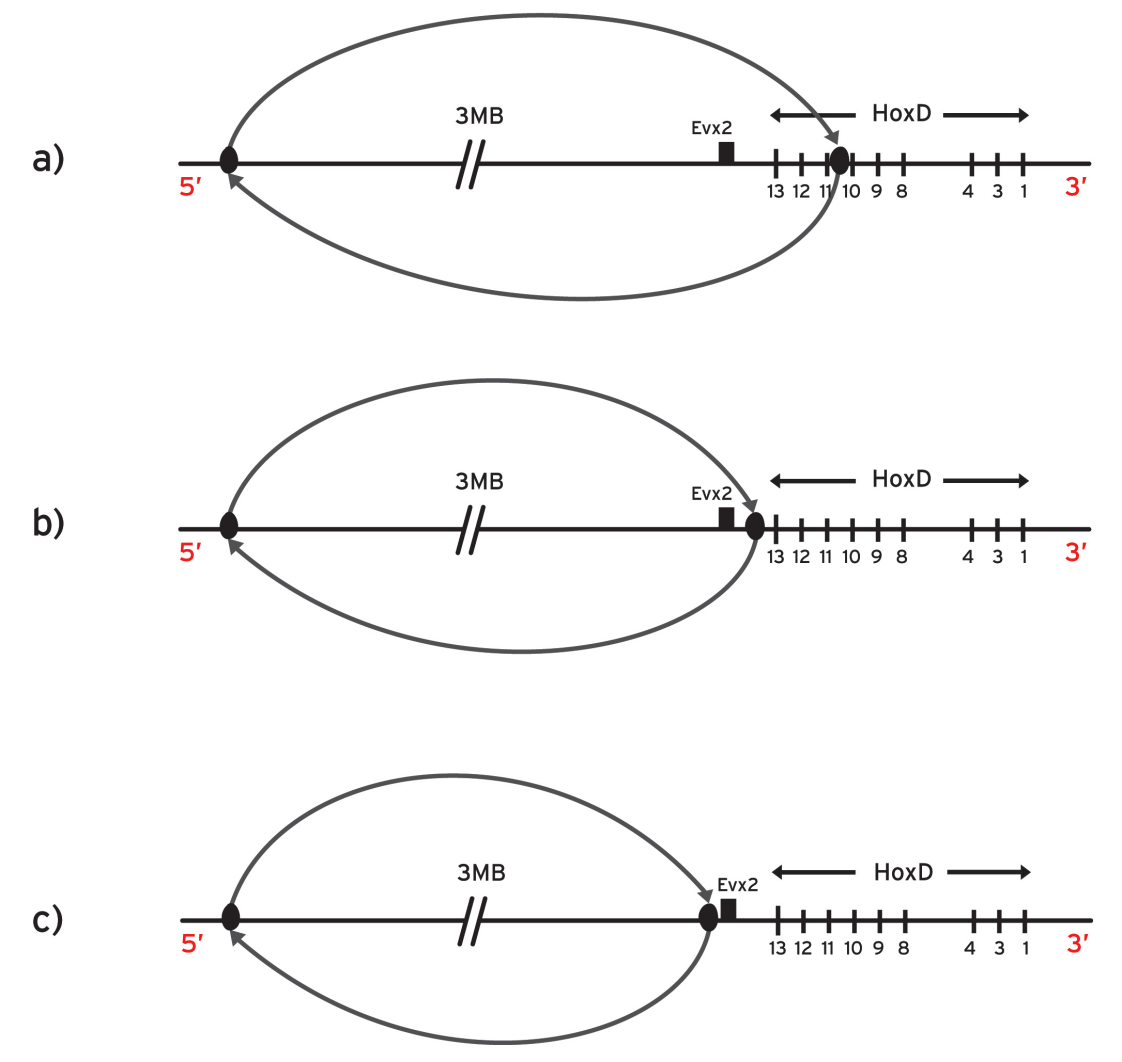
**Posterior *Hoxd* splittings**. **a**) The posterior sub-cluster is split and inverted 3 megabases centromeric to the breakpoint which is located between *Hoxd10* and *Hoxd11*. The fixed end of the spring is removed and consequently the spring can be expanded with smaller than normally forces. **b**) Large inversion centromeric to the whole *HoxD* cluster. *Evx2* is included in the genetic inversion. **c**) Large inversion centromeric to the *HoxD* cluster. *Evx2* is not included in the genetic inversion.

In some splitting experiments the posterior part of the *HoxD* cluster was removed and inverted 3Mb away in the centromeric side (Fig. **5a**) [9]. This extended inversion is thought to remove the inhibitory landscape influence on the *HoxD* cluster [27]. 

At the early stages (E8) the remaining anterior *Hoxd* genes showed premature overexpression in the trunk while the expression of the removed and inverted posterior *Hoxd *genes completely disappeared at these early stages. According to a biomolecular model these observations were attributed to the removal of the centromeric repressing influence on the cluster (‘landscape effect’) [9, 27]. 

Along the same line of thought, the above landscape effect was considered responsible for the results of the following inversion where the centromeric neighborhood was separated from the entire *HoxD* cluster (Fig. **5b**): the splitting is targeted at the intergenic region between *Hoxd13* and *Evx2* while the posterior split is 3Mb apart. As a result the expressions of the posterior genes *Hoxd13-Hoxd10* are prematurely up-regulated at stage E9 [27]. 

The biophysical model proposes a quite different explanation of the above results [15, 16]: in the above posterior splitting experiments (Fig. **5a,b**), the fixed posterior end of the cluster is removed. Consequently, the representative elastic spring becomes loose at both its ends. This makes the DNA fiber susceptible to decondensation and translocation with abnormally smaller forces. Therefore, the mutant *Hoxd *expressions should be prematurely anteriorized in agreement with the data. (See below a proposal to test further the spring-like behavior of the DNA fiber).

## NOISE-INDUCED PERTURBATIONS AND THEIR EVOLUTIONARY IMPLICATIONS

In order to further explore the consequences of the biophysical model we take into account an observation verified in several cases from a variety of taxonomic groups: Animals with a more complex body structure are always characterized by a more compact structure of their *Hox* gene cluster, as opposed to less dense clusters which appear in simpler organisms [2]. Note that in many invertebrates the *Hox *complexes are disorganized or disintegrated. Nevertheless even these *Hox *clusters maintain a kind of spatial collinearity [5]. 

As mentioned above, compact *Hox* gene clustering is correlated to temporal collinearity [24]. Furthermore, when temporal collinearity is not observed the *Hox* clusters tend to be broken or dispersed. By a simple numerical experiment, we now show that the biophysical model is compatible with the above observation and it provides a straightforward way to understand why compact *Hox* gene clusters are more suitable for conveying the necessary positional information for the construction of a more complex body plan. 

The genomic structures which we will compare here are *Hox* clusters from (i) mouse and (ii) amphioxus genomes (a compact and a relatively loose gene concatenation, respectively). We have not included in the comparison structures like the ones met in *Drosophila*, where HOM gene clustering is much looser and additionally is split into two sub-clusters. This is because direct comparison between continuous and split gene clusters is not straightforward.

In the following developmental scenario, sequential exposure to transcription of individual genes is assumed to produce morphologically acting proteins which lead to the formation of a body plan of sequentially appearing phenotypic units (somites, rhombomeres, segments, or other primary body structures). In the present simple implementation, without loss of generality, we assume that the temporal intervals between the consecutive activation of *Hox* genes of the same cluster ideally lead to segments of equal lengths. Then, we perturb this system with environmental noise, which affects the individual segment lengths. We assess the suitability of the geometry characterizing each *Hox* gene cluster to be carrier of positional information, by computing the variance of the resulting unequal segment lengths. 

More specifically: we model the sequence of morphogenetic events, assuming that once the *Hox* gene ***H_i_*** is exposed to transcription (and then formation of its developmentally active product), a series of morphogenetic events starts. This leads to the formation of the corresponding segment, until the activation of the next *Hox* gene ***H_i+1_***, which will give rise to the formation of the next segment. It is then plausible to assume that (at a first approximation) the time of ***H_i_*** functioning is proportional to the distance on the DNA chain between ***H_i _***and ***H_i+1_***. We call this distance ***d(i,i+1), i = *1, ... 13**. Depending on the time of exposure and functioning of each *Hox* gene, the environmental noise will act on the Factor of Phenotypic Realization ***f(i,i+1) ***(FPR), which "translates" the *Hox* gene coding information onto a phenotype trait. In the simple implementation of the ideas described herein, the factors of phenotypic realization obtain values leading to the formation of segments of equal lengths in the ideal (unperturbed) case. The larger the distance between two consecutive *Hox* genes ***H_i_** , **H_i+1_*** is, the longer the time available for the production of the phenotypic characteristic of the first one, ***H_i_** ,* will be, thus resulting into a higher impact of "environmental noise" during this process. This one-to-one translation of the phenotypic traits holds under the assumption that the amplitude distribution of environmental noise is statistically stable in time. Subsequently, we can conjecture that lengthy and irregular *Hox* gene clusters will produce a higher fuzziness in the reproduction of a given body plan. Thus, in view of the biophysical model assumptions, evolution is expected to promote compact and regular *Hox* gene cluster structures if a more complex and still functional body plan is required. 

In order to test the biophysical model conjecture, given the aforementioned correlations found between the complexity of the body plan and the compactness of *Hox* gene cluster [2], we use the distribution of distances separating consecutive *Hox* genes from two model organisms: (i) Mouse (*HoxD* cluster), as a typical example of vertebrates, exemplifying the relatively recent evolution of vertebrate genomes [2]; (ii) The cephalochordate amphioxus taken as a characteristic case of a less complicated body plan. Note that the amphioxus chordate genome appears to be a good surrogate for the ancestral genome of vertebrates [28]. The gene distances ***d(i,i+1) ***are given in (Table **1**). For each *Hox* gene ***i ***the distance ***d(i,i+1) ***to its successive one is given in the 2^nd^ and 3^rd^ column for mouse and amphioxus, respectively. In the 4^th^ column, from the genome of amphioxus are retained only distances between the *Hox* genes which are present in the mouse genome. This “modified amphioxus *Hox* cluster” will serve for an additional comparison with the mouse *Hox* cluster, keeping the same number of *Hox* genes, even though the former no more corresponds to a real animal. The means and variances are also given in the same table. The genomic distances (in thousands of base pairs, Kbps) are taken from references [2, 29, 30].

In the ideal case, where no environmental noise is present, all *Hox* genes would produce segments to which we assign the arbitrary constant length ***l***. Then the FPR ***f(i,i+1***) is defined as:

(2) ***f(i,i+1) = l / d(i,i+1)***

The meaning of (2) is that the *Hox* gene ***H_i _***acts for a duration proportional to ***d(i,i+1)***, in order to produce a segment length ***l(i,i+1) = l = **constant*, equal for all ***i***. An arbitrary value ***l = ***100 was assigned to the phenotypic trait for convenience in the calculations. In our numerical experiment, during the processing time, the FPRs are perturbed by factors which are assumed to obey a stochastic description [31, 32]. *In vivo*, the stochastic perturbations may be due to external (environmental) or internal noise. We assume that this perturbation is quite small, of the order of 5-10% of the average value of the FPRs. The noise-perturbed FPRs are denoted by ***f'(i,i+1) ***and give rise to phenotypical characteristics (here, segment lengths) ***l'(i,i+1) ***as

(3) ***l'(i,i+1) = f' (i,i+1) d(i,i+1)***

**Fig. (6):**
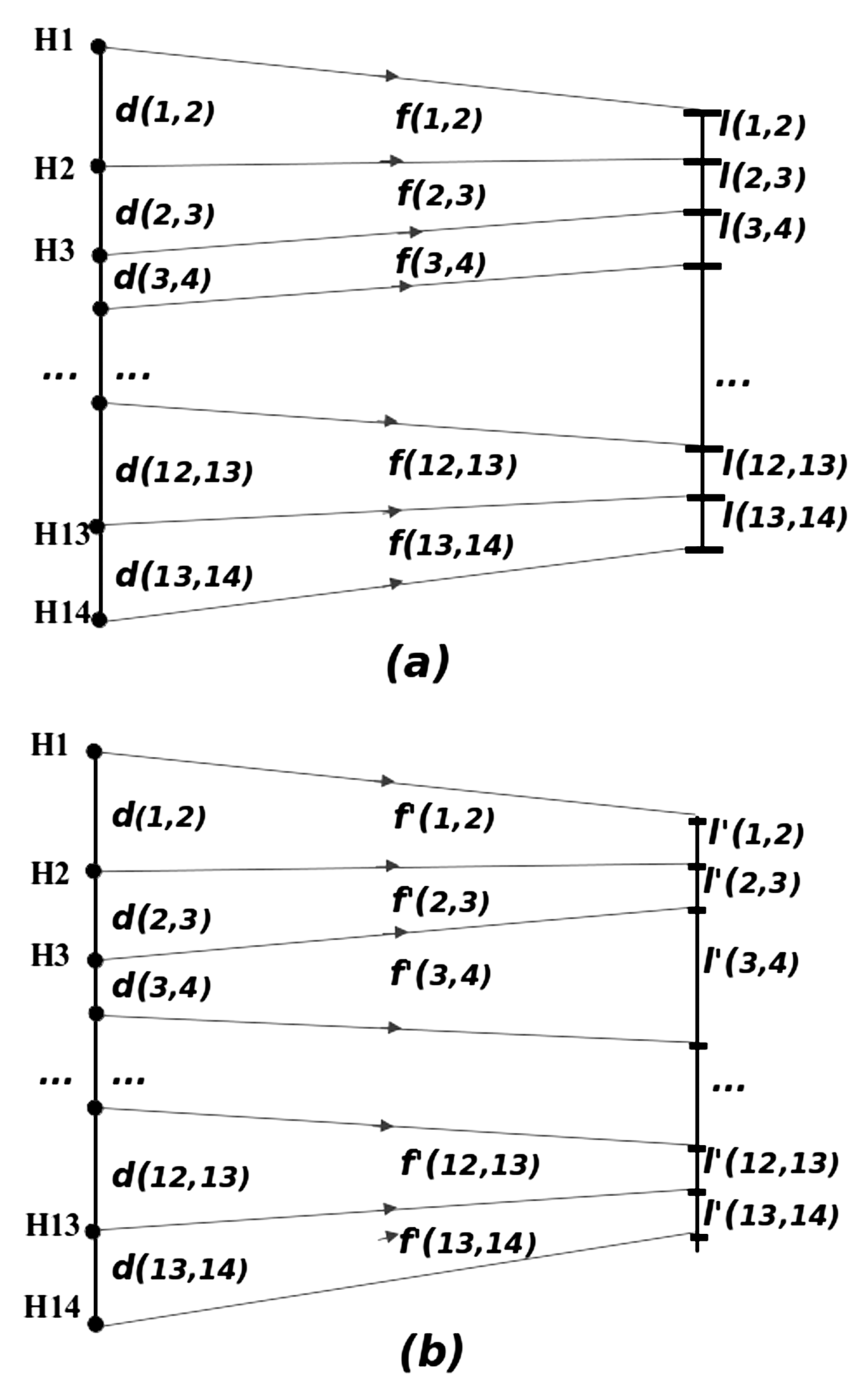
**Unperturbed and perturbed phenotypic traits reflecting the *Hox* gene organization**. Schematic representation for the transformation between (**a**) genomic and unperturbed phenotypic segment lengths and (**b**) genomic and perturbed phenotypic segment lengths.

The variance in the values of*** l'(i,i+1) ***is expected to reflect qualitatively the variations in the phenotypic characteristics observed. Schematically, this process is given in (Fig. **6a,b**). Here, the positions of genes on the DNA thread are figuratively shown with black bullets while the borders between phenotypic segments are marked by dashes. The primed quantities correspond to perturbed variables, while the non-primed ones to the unperturbed.

As a working example we apply Gaussian white noise [32] on the FPRs resulting from formula (2). We assume noise variance 5% and mean 0 for mouse, amphioxus and “amphioxus modified”. The Gaussian-disturbed phenotypical characteristics, as averaged over 250 realisations in all examined cases, are given in (Table **2**).

From (Table **2**), it is evident that when the FPR distributions are perturbed by the same white noise, the variance in the case of an amphioxus-type *Hox* gene structure (loose and irregular, typical of non-vertebrates) is much more pronounced than in the case of a vertebrate-like *Hox* gene structure (mouse). The picture remains the same (in fact, variance is still higher) in the “modified amphioxus” case, where the considered *Hox* genes are only the ones found in the mouse *HoxD* cluster.

In the above simple numerical experiments, according to the biophysical model, the essential features of the *Hox* gene cluster activation are included. It is clearly shown that the observation correlating body complexity with the compactness and regularity of the *Hox* gene structure [2] is compatible with the influence of weak environmental noise. Alternatively, looser body plans tolerate higher levels of environmental noise during development. This is demonstrated for the cluster structure geometry of two characteristic cases, of a vertebrate and a chordate.

## PREDICTIONS AND CONCLUSIONS

1. Consider for the mutant *Hoxd11* the above Case 1 for del(8-10) and the corresponding extruded fiber E(8-10); similarly, Case 2 for del(i-8-10) and the corresponding extruded fiber E(i-8-10). Consider furthermore E(i) the extruded fiber where only the intergenic region “i” is deleted. The biophysical model predicts that for del(i) the mutant *Hoxd11 *expression should be anteriorized [26]. This is a biophysical model prediction worth testing. Such a behavior is not expected according to the biomolecular models [9].

2. In order to test the ‘landscape effect’ and the biomolecular models, the following experiment was proposed [16]: the large centromeric inversion should not include the small intergenic region between *Evx2 *and *Hoxd13 *(Fig. **5c**). For this experiment the model predictions are divergent: according to the biomolecular model, the expressions of the posterior genes *Hoxd13-Hoxd10 *will be comparable to the mutant prematurely anteriorized expressions of the inversion of (Fig. **5c**) [16, 26]. In contrast, the biophysical model predicts that the* Hoxd13-Hoxd10* expressions will be comparable to the *wt* expressions since the small region between *Evx2* and *Hoxd13* (location of the fixed end of the spring) remains intact [16]. This experiment could help us distinguish which of the two models (if any at all) is compatible with the data. 

The experimental results explained above do not exhaust the possible applications of the biophysical model. For instance this model satisfactorily reproduces [14, 15] the delayed and posteriorized *Hoxd* expressions in the limbs of mutant embryos with anterior duplications [8]. Simirarly were explained [14, 15] the *Hoxb1 *transposition experiments into the *HoxD *cluster [33]. 

The biophysical model answers the question whether the physical separation of active from non-active *Hox* genes ‘underlies collinear activation or is a consequence of it’ [10]: from the present study it is clear that both physical separation of *Hox* genes and their collinear activation are indispensable and non-separable elements of a single activation mechanism. This mechanism, based on the application of physical forces, underlies all molecular processes participating in the expression of clustered *Hox* genes. The demonstration that *Hox* gene expression is tightly connected to fiber gene translocations supports the hypothesis of physical forces. After all, any movement from place to place is caused by the application of some force. The recent findings render unavoidable the idea of physical forces being involved in *Hox* gene collinearity.

It is surprising that a simple mechanism like the biophysical model can satisfactorily explain such a wide range of phenomena and so complex experimental results. The speculation that physical principles approximated by the simple eq.(1) might be involved probably reflects some inherent truth hidden in this model.

Finally, the well known dialectic triad of reasoning (Thesis- Antithesis- Synthesis) could be applicable to the collinearity problem: the thesis (Physical Principles) and the anthithesis (Biomolecular Principles) lead to a synthesis (Physical-Biomolecular Cooperation). Thus, the biophysical model underlies the integrated process of *Hox *gene collinearity by determining the temporal and spatial trigger of gene activation. Then, the biomolecular processes regulate the subsequent stages of gene expression.

## Figures and Tables

**Table 1.** The Distances *d(i,i+1)* in Kbps Between *Hox* Genes for the Mouse and Amphioxus

*Hox* Genes Numbers	Intergenic Distances * d(i,i+1) *in Mouse	Intergenic Distances* d(i,i+1) * in Amphioxus	Intergenic Distances * d(i,i+1) *in “Modified Amphioxus”
14			
13		13-14: **62**	13-14: **62**
12	12-13: **6**	12-13: **11**	12-13: **11**
11	11-12: **5**	11-12: **21**	11-12: **21**
10	10-11: **10**	10-11: **32**	10-11: **32**
9	9-10: **6**	9-10: **100**	9-10: **100**
8	8-9: **10**	8-9: **16**	8-9: **16**
7		7-8: **21**	
6		6-7: **42**	
5		5-6: **11**	
4	4-8: **26**	4-5: **63**	4-8: **137**
3	3-4: **16**	3-4: **52**	3-4: **52**
2		2-3: **5**	
1	1-3: **15**	1-2: **10**	1-3: **15**
Total	94	446	446
Mean	11.750	34.308	49.556
Variance	43.686	731.444	1698.025

**Table 2.** The Mean and Variance of the FPR Distribution After Applying Gaussian White Noise. 250 Realizations Are Considered For Each Organism

Organism/Characteristics	Mean	Variance
Mouse	99.745	8.857
Amphioxus	99.772	21.321
Amphioxus modified	99.077	24.511
